# A decision analysis comparing three strategies for peritoneal lavage cytology testing in staging of gastric cancer in China

**DOI:** 10.1002/cam4.3518

**Published:** 2020-10-13

**Authors:** Qifei He, Jinyi Zhu, Anqiang Wang, Ke Ji, Xin Ji, Ji Zhang, Xiaojiang Wu, Xia Li, Zhaode Bu, Jiafu Ji

**Affiliations:** ^1^ Department of Gastrointestinal Surgery Key Laboratory of Carcinogenesis and Translational Research (Ministry of Education) Peking University Cancer Hospital & Institute Beijing China; ^2^ Center for Health Decision Science Harvard T.H. Chan School of Public Health Boston MA USA; ^3^ Department of Medical Epidemiology and Biostatistics Karolinska Institutet Stockholm Sweden

**Keywords:** cost‐effectiveness, cytology, decision analysis, gastric cancer

## Abstract

**Background:**

Positive peritoneal cytology (PCY) indicates metastasis (M1) in gastric cancer (GC) patients; both the American and Chinese guidelines recommend laparoscopic peritoneal lavage (LPL) for cytology. However, relatively high costs impair the widespread use of LPL in some resource‐limited regions in China, and the cost‐effectiveness of PCY testing remains unclear. Therefore, we performed a decision analysis to evaluate the cost‐effectiveness of PCY testing by comparing the guideline‐recommended intraoperative LPL, a newly proposed preoperative percutaneous peritoneal lavage (PPL), and a third strategy of exploratory laparotomy with no cytology testing (ELNC) among GC patients.

**Methods:**

We developed a decision‐analytic Markov model of the aforementioned three strategies for a hypothetical cohort of GC patients with curative intent after initial imaging, from the perspective of Chinese society. We estimated costs, quality‐adjusted life years (QALYs), and incremental cost‐effectiveness ratios (ICERs) as primary outcomes; we also conducted one‐way and probabilistic sensitivity analyses to investigate the model's robustness.

**Results:**

We found that ELNC was dominated (i.e., more expensive and less effective) by PPL and LPL. LPL was the most cost‐effective method with an ICER of US$17,200/QALY compared to PPL, which was below the Chinese willingness‐to‐pay (WTP) threshold of US$29,313 per QALY gained. In sensitivity analyses, PPL was more likely to be cost‐effective with a lower WTP threshold.

**Conclusions:**

Cytology testing through either LPL or PPL was less expensive and more effective than ELNC among GC patients. Moreover, LPL was the most cost‐effective modality at the current WTP threshold, while PPL could potentially be cost‐effective in lower‐income areas.

## Graphical abstract

This is the first decision analysis evaluating the cost‐effectiveness of peritoneal cytology tests among gastric cancer patients. We found performing cytology testing during the gastric cancer staging dominated (i.e., less expensive and more effective) no cytology testing among patients with curative intent, and the guideline‐recommended laparoscopy with cytology was the most cost‐effective modality at the current willing‐to‐pay threshold in China.

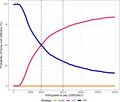

## 1 INTRODUCTION

Gastric cancer (GC) is the third leading cause of cancer death worldwide and ranks second in China.^1^, ^2^ Surgery is the primary treatment option for patients with localized advanced gastric cancer (AGC), but only in the absence of non‐curative factors can radical resection be achieved. Positive peritoneal cytology (PCY), even in the absence of visible peritoneal implants, is considered as metastasis (M1) disease, in which case surgery would not be recommended as initial treatment according to the National Comprehensive Cancer Network (NCCN) guidelines.^3^ Therefore, early and accurate detection of PCY is critical in the management algorithm.

Both the NCCN and the Chinese Society of Clinical Oncology (CSCO) guidelines recommend performing laparoscopy with cytology to detect radiographically occult peritoneal metastases (OPM).^3^, ^4^ However, a nationwide survey showed that both staging laparoscopy(SL) and intraoperative peritoneal lavage (IPL) for cytology are not practiced routinely in most centers due to high costs and operational requirements such as general anesthesia and formal operating rooms.^5^ Exploratory laparotomy with no cytology testing (ELNC) is a more common way to evaluate the resectability or curability of AGC in China, especially in some low‐income areas where laparoscopy may be neither feasible nor affordable.^6^ Furthermore, according to a retrospective study, only 17% had supplementary IPL among those who had undergone SL in China.^7^ Hence, there is a contradiction between guideline recommendation and clinic practice in some areas.

Besides IPL during SL or exploratory laparotomy, percutaneous peritoneal lavage, a safe and effective method to determine the likelihood of peritoneal penetration in trauma settings,^8^ was proposed as an alternative of preoperative diagnosis for peritoneal lavage cytology in AGC cases by Makino, Pak, and James.^9^, ^10^, ^11^ Compared with the intraoperative laparoscopic peritoneal lavage (LPL), the preoperative percutaneous peritoneal lavage (PPL) is easier and cheaper to perform.^9^ However, the sensitivity of PPL is inferior to the LPL.^9^, ^10^, ^11^ Furthermore, PPL provides less information on resectability than LPL as LPL could visually inspect the peritoneal cavity when collecting cytology specimen.

Therefore, more evidence, including the accuracy of testing, utilities, and costs, is needed to synthesize to evaluate the payoff of PCY testing. To the best of our knowledge, no previous study has reported the cost‐effectiveness of PCY testing among GC patients. We aim to evaluate the cost‐effectiveness of PCY testing by comparing two active cytology testing strategies of the intraoperative LPL (a guideline recommendation) and the preoperative PPL (a newly proposed testing) against the third strategy of ELNC (a common practice in rural regions) for GC patients with curative intent.

## 2 METHODS

### 2.1 Model structure

We compared the three PCY testing strategies and examined their subsequent treatment in terms of costs and health outcomes from a Chinese societal perspective. Our model simulated a hypothetical population of age 56 (i.e., the average age in the CLASSIC trial),^12^ who was assumed to have no history of chemotherapy and have been diagnosed with locally AGC, however, with no radiographic metastases at the start of the model. That is, all patients were assumed with curative intent based on initial imaging but could suffer from OPM as their PCY status was unknown. That is, all patients were assumed with curative intent based on initial imaging but could suffer from occult peritoneal metastases (OPM) as their PCY status was unknown.

**FIGURE 1:**
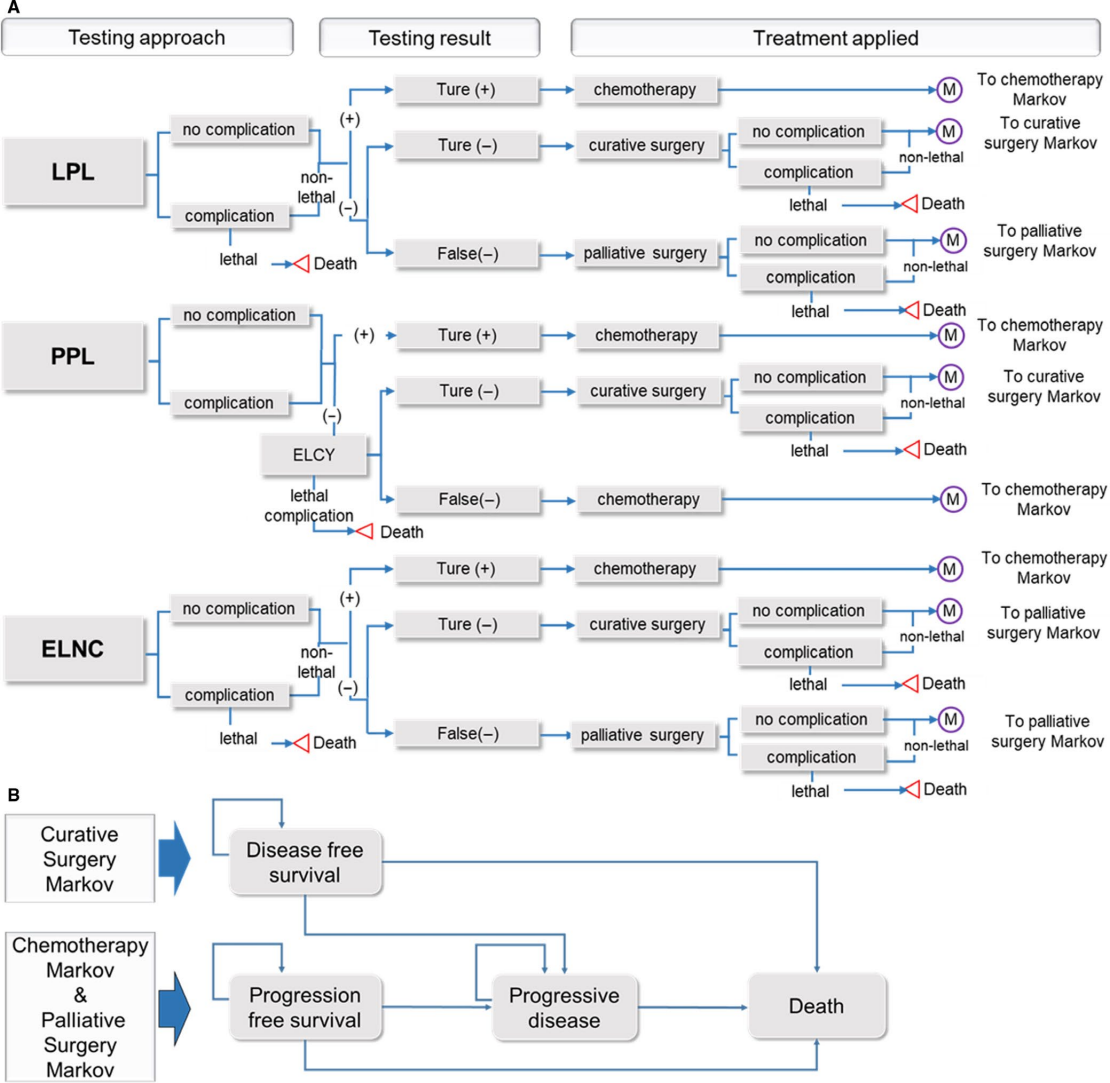
Markov‐Decision analysis model layout. A decision tree containing Markov models shows three strategies for individuals with gastric cancer who are assumed as radiographically occult metastases disease with curative intent. Panel A. illustrates the three strategies to detect peritoneal cytology: LPL, PPL, and ELNC. Panel B shows the Markov model comprises three health states of disease/ progression‐free survival, progressive disease, and death. Abbreviations: ELCY, exploratory laparotomy with cytology; ELNC, exploratory laparotomy with no cytology; LPL, laparoscopic peritoneal lavage; PPL, percutaneous peritoneal lavage

A Markov model (Figure 1) was established using TreeAge Pro 2019 (TreeAge Software Inc.) to evaluate the cost‐effectiveness of three strategies: LPL, PPL, and ELNC followed by chemotherapy/surgery. The full pathways were modeled for the patients from the start of the PCY testing, through treatments of chemotherapy, surgery, or palliative therapy, until death or the end of the 40‐years simulation period. The testing results determined the subsequent treatments according to the NCCN and the CSCO guidelines.^3^, ^4^ Specifically, patients who tested positive for PCY (i.e., positive cytology with or without visible peritoneal implants, CY1PX) received chemotherapy and those who tested negative received surgery (Figure 1 Panel A). Curative surgery can be achieved only for those with true negative results (i.e., CY0P0), while non‐curative palliative surgery was performed for those with false‐negative results (i.e., CY1PX and CY0PX). Besides, we assumed that all patients with a positive result had metastatic disease, given the specificity of these tests was estimated to be close to 100% in previous studies.^9^, ^10^, ^11^, ^13^ Therefore, false‐positive PCY cases were not considered in our model. Patients with a negative result of PPL would undergo an exploratory laparotomy with intraoperative cytology, according to Makino's protocol.^9^ In the following pathway of PPL, we assumed that the laparotomy with a second cytology testing intraoperatively could detect all the OPM that were missed during the PPL. In the pathway of ELNC, we assumed that exploratory laparotomy could find all the visible peritoneal implants. Considering radiographically occult peritoneal metastases refer to positive cytology and visible peritoneal implants; thus, the false‐negative possibility of the strategy of ELNC equals the prevalence of CY1P0 (i.e., positive cytology without visible peritoneal implants). As LPL and PPL are minimally invasive techniques compared with laparotomy, the discrepancies of these techniques in complication rates were also considered. We assumed nonfatal complications would increase cost but result in no long‐term influence on utility. Fatal complications were assumed not to be observed in the PPL subtree, based on previous reports.^8^, ^9^, ^10^ In the decision model, data inputs of transition probabilities were derived from published studies (Table 1).

**TABLE 1:** Key model variables

Parameter	Base‐case value	Sensitivity analysis range	Distribution	Source(s)
Probability				
Pretest probability of OPM	23.4%	8.5‐59.6%	Beta	Mezhir & Leake^18^, ^19^
Sensitivity of PPL for cytology metastases^a^	75.9%	65.1‐84.2%	Beta	Makino, Pak & James^9^, ^10^, ^11^
Sensitivity of LPL for OPM	84.6%	74.7‐91.8%	Beta	Ramos^13^
Specificity of testing for OPM^c^	100%		Beta	Makino, Pak, James & Ramos^9^, ^10^, ^11^, ^13^
Probability of CY1P0^a^	6.4%	4.5‐8.7%	Beta	Mezhir, Bando, Lee & Kuramoto^18^, ^20^, ^21^, ^22^
Probability of PPL complication^b^	0.8%	0.4‐1.2%	Beta	James^23^
Probability of LPL complication^b^	2.2%	0‐5%	Beta	Muntean^24^
Probability of EL complication^b^	6%	3‐9%	Beta	Smith^25^
Probability of gastrectomy complication^a^	21.6%	19.4‐23.9%	Beta	Wu, Papenfuss & Martin^26^, ^27^, ^28^
Perioperative mortality of LPL	0.07%	0‐0.13%	Beta	Muntean, Adamek^24^, ^29^
Perioperative mortality of EL	1.5%	0‐3%	Beta	Burke, Smith^22^, ^25^
Perioperative mortality of gastrectomy^†^	2.7%	0.3‐7.5%	Beta	Wu, Papenfuss & Martin^26^, ^27^, ^28^
**Cost**				
PPL	246	123‐369.	Gamma	Calculated
LPL	2213	1107.5‐3319.5	Gamma	Calculated
EL	2065	1032.5‐3097.5	Gamma	Calculated
Surgery Annual direct medical cost	9,617	3521‐16289	Gamma	Yang^30^
Annual indirect cost	664	353‐1217	Gamma	
Annual direct nonmedical cost	320	195‐541	Gamma	
Adjuvant chemotherapy per cycle	2635	2208‐3063	Gamma	He^31^
Chemotherapy Annual direct medical cost	3,697	1802‐7411	Gamma	Yang^30^
Annual indirect cost	1189	1008‐1906	Gamma	
Annual direct nonmedical cost	370	359‐411	Gamma	
Palliative therapy Annual direct medical cost	3109	1348‐9636	Gamma	Yang^30^
Annual indirect cost	855	785‐868	Gamma	
Annual direct nonmedical cost	367	349‐371	Gamma	
Folds of complication cost versus surgery cost^b^	3	1.5‐4.5	Normal	Luke^32^
**Utility**				
Adjuvant chemotherapy after surgery (<6 m)	0.68	0.56‐0.76	Beta	Tan^33^
Postgastrectomy state with the accomplishment of adjuvant chemotherapy (>6 m)	0.81	0.65‐0.97	Beta	Tan^33^
Metastasis GC with palliative surgery plus chemotherapy	0.54	0.52‐0.56	Beta	Li^34^
Metastasis GC with chemotherapy	0.66	0.58‐0.73	Beta	Li^34^
Recurrent or progressive state with palliative therapy	0.40	0.10‐0.69	Beta	Lee^35^
**Other parameters**				
Discounted rate^c^	3%		—	Liu^36^

Abbreviations: CY1P0, positive cytology without visible peritoneal implants; EL, exploratory laparotomy; GC, gastric cancer; LPL, laparoscopic peritoneal lavage; OPM, occult peritoneal metastases; PPL, percutaneous peritoneal lavage.

^a^ Derived from the random‐effect meta‐analysis.

^b^ Range estimated as 50‐150% of base case value.

^c^ Not assessed in the sensitivity analysis.

We created three Markov models corresponding to the above three treatments: curative resection, palliative resection, and chemotherapy (Figure 1B). Each Markov model comprises three health states of "disease‐free survival" (DFS) or "progression‐free survival" (PFS), "progressive disease" (PD), and "death," in which DFS and PFS are used for curative resection Markov model and non‐curative palliative resection/ chemotherapy Markov model, respectively (Figure 1B). The Markov models captured monthly outcomes and costs of continued medical treatment. We assumed, upon entering the Markov pathway of chemotherapy, patients with positive metastases were not able to switch to negative or terminate treatments unless progression occurs. Transition probabilities among these various health states were derived from the Chinese life tables and randomized controlled trials (RCTs) that were the most similar to the population in our model.^12^, ^14^, ^15^, ^16^ PFS and OS from these clinical trials listed in Table 2 were used for calculating the Markov state transition probabilities. When calculating the time‐dependent probabilities during each Markov model cycle, we first extracted and digitalized the survival probabilities from the corresponding Kaplan‐Meier plots reported by the previous clinic trials using Engauge Digitizer version 10.8 software. Next, we compared four commonly used parametric models to fit the digitalized Kaplan‐Meier curves, which assumed Weibull, exponential, log‐logistic, and log‐normal distributions. Third, we chose the most reasonable survival distribution function based on clinical rationality, visual fit, and statistical goodness‐of‐fit using Bayesian information criteria (BIC) and Akaike information criteria (AIC) (Figure S1 and Table S1). Weibull distribution was selected for the transition probability from DFS to DFS/PD and PD to death, while the log‐logistic distribution was chosen to calculate transition probability from PFS to PFS/PD. The mortality in the DFS or PFS state was derived from the age‐related mortality rate in Chinese life tables.^15^, ^17^ The additional model assumptions and details of model selection are provided in Supporting Information.

**TABLE 2:** Information on clinical trials and survival model parameters

Markov status transition	Clinical Trial	Optimal model^a^	Parameter value^b^
DFS to PD of curative surgery strategy for true negative PCY result	DFS of gastrectomy with adjuvant chemotherapy arm of CLASSIC trial	Weibull	*λ* = 0.0132, *γ* = 0.8454
PFS to PD of palliative surgery strategy for false‐negative PCY result	PFS of chemotherapy plus palliative gastrectomy arm of REGATTA trial	log‐logistic	*a* = 0.0142, *b* = 2.0360
PFS to PD of chemotherapy strategy for true positive PCY result	PFS of chemotherapy arm of REGATTA trial	log‐logistic	*a* = 0.0069 *b* = 2.3024
PD to death	OS of docetaxel arm of COUGAR−02 study	Weibull	*λ* = 0.0654, *γ* = 1.3663

Abbreviations: DFS, disease‐free survival; PCY, peritoneal cytology; PFS, progression‐free survival; PD, progressive disease; OS, overall survival.

^a^ The selection process of the optimal distribution is seen in the Supporting Information file.

^b^ The survival function of Weibull and log‐logistic distribution is exp(−*λt^γ^*) and 1/(1 + *at^b^*), respectively.

### 2.2 Costs and utility

Costs involved direct medical cost, direct nonmedical cost, and indirect cost of GC patients. Annual direct and indirect costs of surgery, chemotherapy, and palliative therapy were obtained from a recent national multicenter survey in China (Table 1).^30^ The average direct costs for patients who underwent PPL, DL, and ELNC were estimated using data from Peking University Cancer Hospital, which is linked to the Beijing's medical insurance information system. The costs of adjuvant chemotherapy, surveillance, and complication were also incorporated. All costs were expressed in RMB values of year 2019 and converted into US dollars at an exchange rate of $1= ¥6.75 as observed in the first quarter of 2019.^37^

Health outcomes were quantified using quality‐adjusted life years (QALYs) (Table 1). Details on selecting utility estimates and costings are provided in the Supplementary Methods. We applied a discount rate of 3% per year to all costs and QALYs.

### 2.3 Outcomes and data analysis

#### 2.3.1 Primary outcome

The primary outcome was the incremental cost‐effectiveness ratio (ICER), which is calculated as incremental costs divided by incremental effectiveness. The ICER was compared to a willingness‐to‐pay (WTP) threshold to determine which approach was cost‐effective. In our base‐case analyses, the WTP threshold was set at US$29,313/QALY, approximately threefold the gross domestic product (GDP) per capita in 2018 China (US$9,770.85) as recommended by the World Health Organization (WHO)‐CHOICE (**CHO**osing **I**nterventions that are **C**ost‐**E**ffective) document.^36^, ^38^, ^39^

#### 2.3.2 Sensitivity analyses

To evaluate the influence of parameter uncertainty on model robustness, we performed both one‐way and probabilistic sensitivity analyses (PSA). The ranges and distributions used are summarized in Table 1. In the one‐way sensitivity analyses (OWSA), we allowed the value of each input to vary within its plausible range keeping the other constant. We then performed threshold analyses on those influential parameters to which the outcome was particularly sensitive. Furthermore, we conducted PSA by varying all variables simultaneously over their respective ranges and distributions in 1,000 Monte Carlo simulations to evaluate the impact of fluctuations across all parameters. The input variables were assumed to follow specific distributions: Gamma distributions were used for costs, whereas beta distributions were used for utilities and probabilities.

## 3 RESULT

### 3.1 Base case analyses

Table 3 shows cost‐effectiveness outcomes for both an undiscounted model and a discounted model. ELNC was dominated (i.e., more expensive and less efficacious) by both PPL and LPL in both models. Compared with the PPL, the incremental costs for each QALY gained for the LPL approach were $17,200 in the discounted model and $12,038 in the undiscounted model, which were less than our prespecified WTP threshold. Thus, our base case analysis demonstrated that LPL was the most cost‐effective strategy at a WTP threshold of $ 29,313 per QALY.

**TABLE 3:** Base case cost‐effectiveness results

	Undiscounted			Discounted^a^		
	Cost (US$)	Effectiveness (QALYs)	ICER ($/QALY)	Cost (US$)	Effectiveness (QALYs)	ICER ($/QALY)
ELNC	24,097	8.03	Dominated	23,738	5.81	Dominated
LPL	24,100	8.15	12,038	23,736	5.90	17,200
PPL	22,901	8.06	Reference	22,515	5.83	Reference

Abbreviations: ELNC, exploratory laparotomy with no cytology; ICER, incremental cost‐effectiveness ratio; LPL, laparoscopic peritoneal lavage; PPL, percutaneous peritoneal lavage; QALY, quality‐adjusted life years.

^a^ Discounted at 3%.

### 3.2 Sensitivity analyses

**FIGURE 2:**
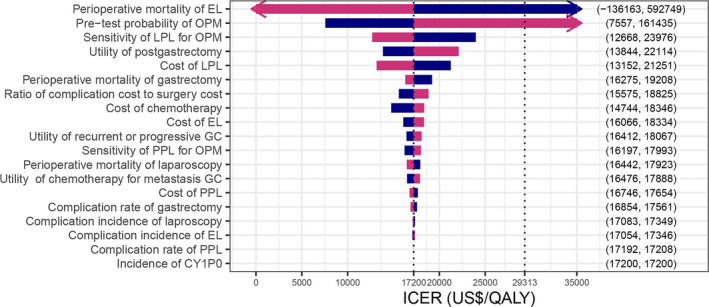
One‐way sensitivity analysis results (ICERs for LPL vs. PPL). Tornado diagram summarizes one‐way sensitivity analyses for the LPL strategy compared with the PPL strategy for the base‐case analysis. Most ICERs were close to the base‐case result (US$17,200 per QALY) as model parameters were varied through plausible ranges, with the exceptions of the perioperative mortality of ELNC and the pretest probability of occult peritoneal metastases. Parameters are shown in descending order of influence on model results. The blue portion and the red portion of the bar, respectively, represent the ICER range when the parameter value is lower and higher than the base‐case result. ICER, incremental cost‐effectiveness ratios; QALY, quality‐adjusted life years; LPL, intraoperative laparoscopic peritoneal lavage; PPL, preoperative percutaneous peritoneal lavage; EL, exploratory laparotomy

**FIGURE 3:**
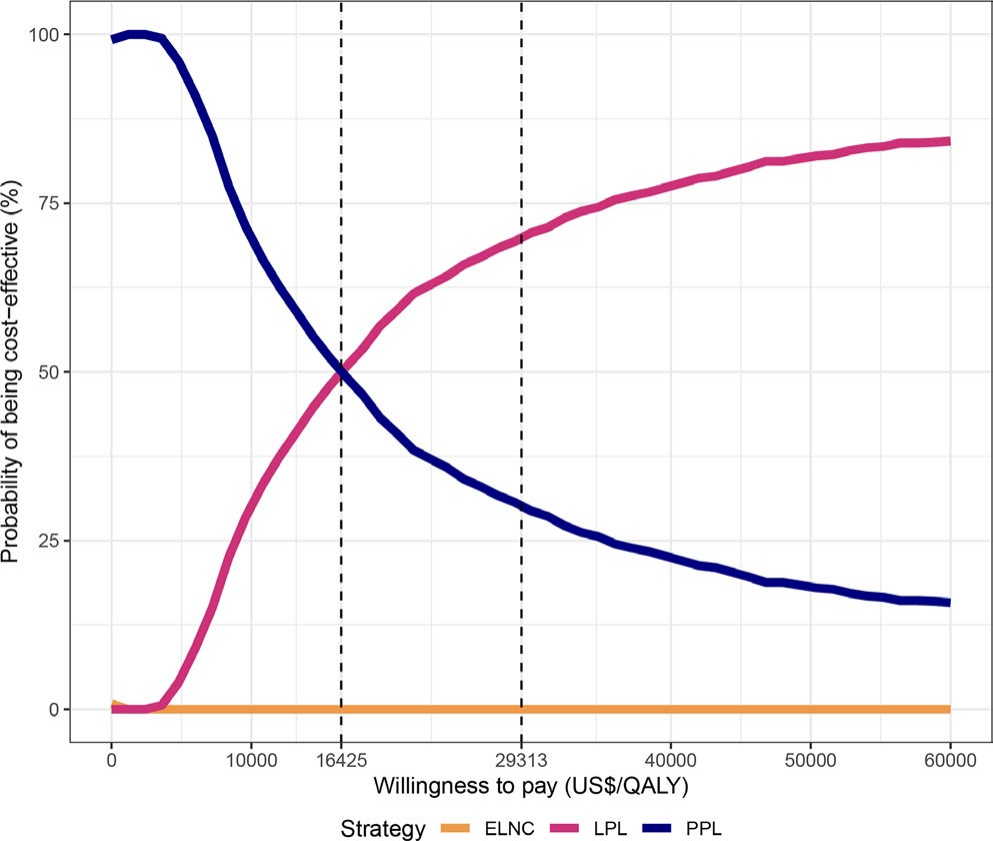
Cost‐effectiveness acceptability curve. The curves illustrate the probability of being cost‐effective for each PCY strategy. The probability that LPL was cost‐effective increased as the WTP threshold went up, while the probability of PPL declined. ELNC had nearly zero probability of being cost‐effective across the WTP threshold spectrum. The probability of cost‐effectiveness was 66.8% for LPL at the prespecified WTP threshold of US$29,313/QALY. The threshold where LPL and PPL have an equal probability of being cost‐effective was US$16,425/QALY. Abbreviations: ELNC, exploratory laparotomy with no cytology; LPL, laparoscopic peritoneal lavage; PPL, percutaneous peritoneal lavage; QALY, quality‐adjusted life years; WTP, willing‐to‐pay

As ELNC strategy was dominated at any WTP threshold, which was further illustrated in the subsequent PSA (Figure 3), OWSA of LPL in comparison with PPL were performed (Figure 2). The results, illustrated with a tornado diagram of ICERs, indicated that the costs, utility weights, and effectiveness of testing had little individual influence on LPL being more cost‐effectiveness at the WTP threshold of $29,313/QALY. The mortality rate of nontherapeutic laparotomy after PPL and the prevalence of radiographically OPM were two of the most influential parameters. We found other input values within their plausible or reported range in Table 1 would not change the result, that is, LPL was the most cost‐effective approach. Threshold analyses (Table S3) show that PPL became cost‐effective at the WTP of $29,313/QALY when the mortality of laparotomy following PPL was less than 1.06%. A higher probability of OPM (greater than 31.03%) would also result in PPL being cost‐effective.

Cost‐effectiveness acceptability curves (Figure 3) illustrate the results of PSA, determining the probability of being cost‐effective for each PCY strategy over a range of WTP thresholds. The probability of cost‐effectiveness was 66.8% for LPL at a WTP threshold of $29,313/QALY. Besides, when the WTP threshold was less than $16,425/QALY, PPL was more likely to be cost‐effective. The strategy of ELNC was unlikely to be cost‐effective compared with PPL and LPL across the WTP threshold spectrum.

## 4 DISCUSSION

We modeled the long‐term cost‐effectiveness following three testing strategies of PCY among GC patients and found that performing PCY testing for potentially curative GC dominated (i.e., less expensive and more effective) non‐testing strategy, through either LPL or PPL. In particular, LPL was the most cost‐effective option at a WTP threshold of approximate the threefold GDP per capita in China. Furthermore, OWSA and PSA suggested PPL had the potential to be cost‐effective under the circumstances with a lower perioperative exploratory laparotomy mortality, a higher incidence of OPM, or a lower WTP threshold.

The payments of GC inpatients in China had increased to approximately 1.5 billion US dollars in 2015, which imposes substantial financial burdens on both GC patients and the health system.^40^ Cost‐effectiveness evidence is necessary to improve the efficiency of health resource allocation. As cytology testing can help surgeons distinguish some of the locally AGC (potentially curatively resectable) from metastatic disease (unresectable), it is important to evaluate the potential health economic impact of the cytology‐involved diagnostic staging methods. A previous meta‐analysis also showed a prognostic benefit of using peritoneal lavage cytology.^41^ Our study suggested that PCY testing was not only effective in terms of higher QALY gains, but also cost‐effective. Our finding that PCY testing was less costly and more effective than those without testing added new health economic evidence to support PCY testing in the diagnostic staging of GC.

Laparoscopy along with cytology of peritoneal washing is the current guideline‐recommended approach to obtain PCY specimen.^3^, ^4^ However, relatively few gastric cancer patients undergo staging laparoscopy before gastrectomy, in both China and the United States.^5^, ^42^ Li et al. even found routine SL is less cost‐effective than laparotomy from a societal perspective in the United States.^34^ These guideline‐conflict findings may partly result from the neglect of the role of PCY in Li's study. As such, our study explicitly included cytology in the SL procedure and compared it with ELNC, so that SL strategy got the added diagnostic accuracy resulting from the supplemented PCY testing. We found that LPL was the most cost‐effective at our prespecified WTP threshold. Furthermore, although the routine LPL is more invasive and costly than PPL, LPL was more cost‐effective as long as the WTP threshold is more than $16,425/QALY, possibly owing to the relatively high sensitivity of LPL in the detection of OPM. Therefore, our finding supports the guideline recommendation of the use of laparoscopy along with cytology of peritoneal lavage from a health economic perspective in China.

PPL is a convenient and attractive approach to detect PCY, which can be performed with local anesthesia and a minimally invasive incision outside the standard operating room. Makino found the cost of PPL is only about one‐ninth of the cost of LPL.^9^ Even though the ratio (1:9) of two procedures’ costs is consistent with our cost data, we found the PPL was not cost‐effective at the current WTP. However, sensitivity analyses suggested that PPL was more likely to be cost‐effective in certain conditions. The acceptability curves show PPL was cost‐effective with a low WTP. There are 34 first‐level administrative divisions (i.e., province) in China. The per‐capita GDP of each province in mainland China varies significantly from US$21,188 in Beijing (a metropolitan city) to US$4,735 in Gansu province (an underdeveloped area). ^5^, ^43^ LPL yielded an ICER of US$16,673 compared to PPL, which exceeded the local WTP threshold if we chose the WTP threshold of three times the per‐capita GDP of Gansu province (US$14,205). Individuals there in low socioeconomic status suffer a higher risk of GC incidence and cancer‐specific mortality than those in high status, probably due to higher rates of H. pylori infection, higher intake of starchy food, and lower access to fresh food and vegetables.^44^ A cost‐effective and affordable testing method is important in GC management. Thus, PPL could be advantageous and worth advocating in diagnostic stating of GC in the resource‐limited settings in China. Besides China, barriers to gastric cancer care are substantial in the rural low‐ and middle‐income countries (LMICs) setting of Central America.^45^ This result might contribute the cancer control in LMICs and promote the establishment of cost‐effective cancer care there. Moreover, our findings were sensitive to the procedural mortality rate of laparotomy and the prevalence of OPM, which implied that PPL could be a feasible procedure in selected patients with a high risk of OPM metastases, or with clinical and surgical improvements of lower mortality of laparotomy.

The strengths of our study include that we used a decision analytical modeling and cost‐effectiveness analysis to resolve a contradiction between guideline recommendation and clinical practice. Clinical practices, especially in developing areas, are sometimes limited by the shortness of infrastructure, technology, and other health resources.^6^ Consequently, some guideline recommendations or innovative treatments cannot be fully carried out and popularized.^5^, ^6^ Our study provided an example of resolving this clinical issue from a health economic perspective. Another strength of our study is that we used a well‐developed decision‐analytic Markov model and synthesized evidence on the prevalence and prognosis of PCY metastases, the accuracy and minimal invasion of testing, and the associated health outcomes and costs. This evidence is especially informative in the absence of the PPL‐related RCTs.

One limitation of our study is that other management algorithms for patients with locally AGC may exist, except for the treatment pathway included in our model. Testing approaches, such as exploratory laparotomy with cytology and laparoscopy without cytology, were not investigated separately. However, these uninvolved strategies have been taken into consideration to some extent. For instance, the effects due to the uncertainty of the costs and health effects were quantified by performing sensitivity analysis. Moreover, as we assumed that patients with positive metastases are not able to convert negative in the Markov model, our model do not incorporate the scenarios of neoadjuvant chemotherapy and conversion surgery. Future studies could explore the possible application of PPL in combination with the neoadjuvant/conversion chemotherapy.

In conclusion, performing cytology testing to stage GC through either LPL or PPL‐dominated ELNC, among Chinese patients with curative intent after initial imaging. Moreover, LPL is the most cost‐effective modality at the current WTP threshold, while PPL could be cost‐effective in areas with a low WTP, in situations with low laparotomic perioperative mortality, and among selected GC patients with a high incidence of OPM. The decision concerning the recommended choice of cytology testing, percutaneous or laparoscopic peritoneal lavage, could be made according to the local socioeconomic status in China. Our study provides evidence on cost‐effectiveness to facilitate clinical decision making of PCY testing and to improve resource allocation efficiency for GC management.

## CONFLICT OF INTEREST

All authors report no potential conflicts of interest.

## AUTHOR CONTRIBUTIONS

**Qifei He:** Conceptualization, formal analysis, funding acquisition, methodology, software, and writing – original draft. **Jinyi Zhu:** Formal analysis, and writing – review and editing. **Anqiang Wang:** Data curation and funding acquisition. **Ke Ji:** Data curation. **Xin Ji, Ji Zhang,** and **Xiaojiang Wu:** Investigation, and validation. **Xia Li:** Writing – review and editing, and visualization. **Zhaode Bu** and **Jiafu Ji:** Conceptualization, funding acquisition, project administration, resources, and supervision. All authors read and approved the final manuscript.

## ETHICS APPROVAL

Not applicable.

## Supporting information

Fig S1

Supplementary Material

## Data Availability

The data that support the findings of this study are available from the corresponding author upon reasonable request.
