# Supplementary figures and images for: A decision analysis comparing three strategies for peritoneal lavage cytology testing in staging of gastric cancer in China
Source: Cancer Med. 2020 Oct 13;9(23):8940–9. doi: 10.1002/cam4.3518 (PMC7724308; doi:10.1002/cam4.3518)

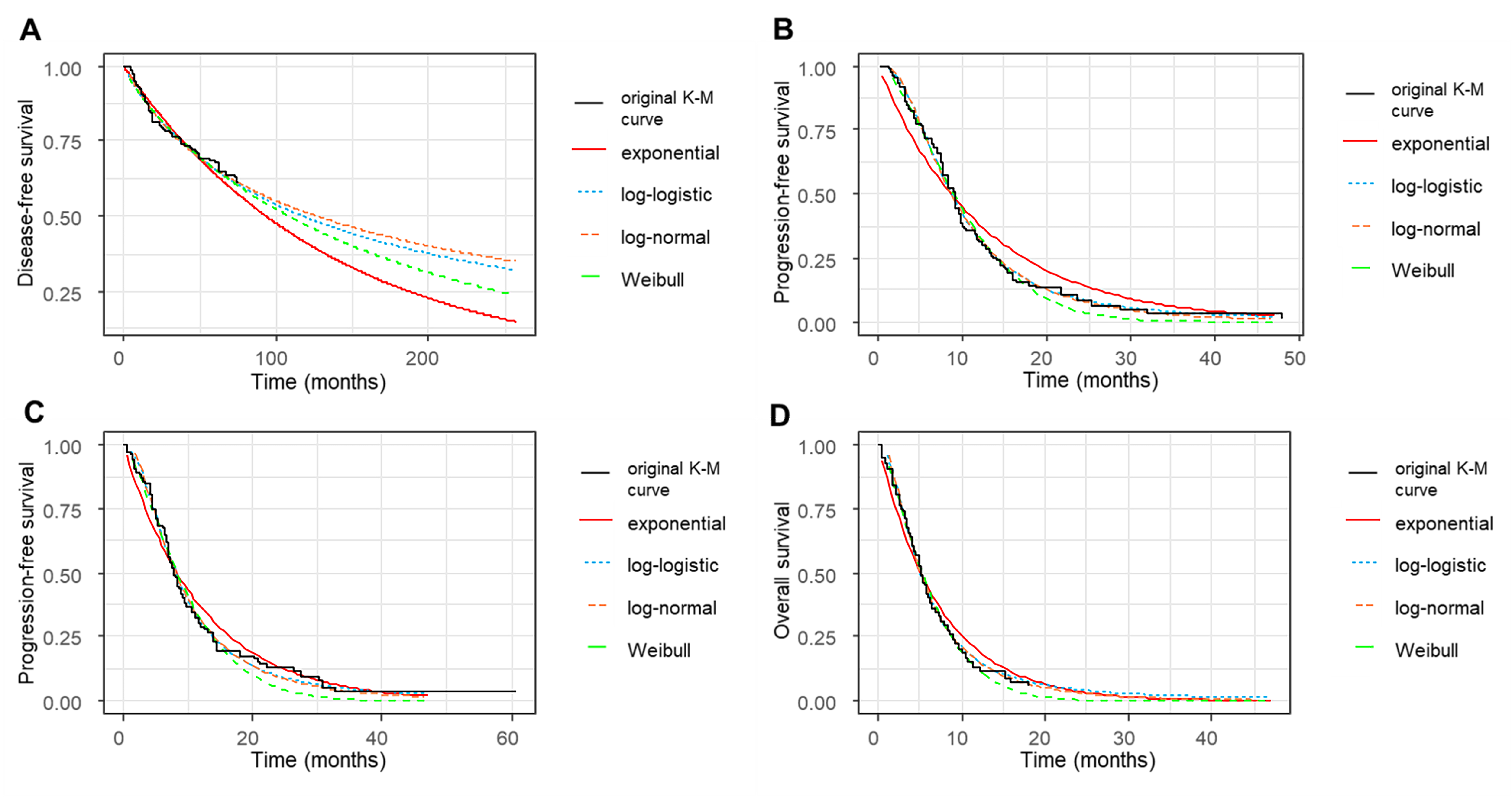

Supplement: Supplementary file 1 — Fig S1 [file CAM4-9-8940-s001.tif]
