# Supplementary material for: A decision analysis comparing three strategies for peritoneal lavage cytology testing in staging of gastric cancer in China
Source: Cancer Med. 2020 Oct 13;9(23):8940–9. doi: 10.1002/cam4.3518 (PMC7724308; doi:10.1002/cam4.3518)
Supplement: Supplementary file 2 — Supplementary Material [file CAM4-9-8940-s002.doc]

**Supporting Information**

Supporting assumptions and details include:

1. Decision tree
   1. Patients with a negative result of preoperative percutaneous peritoneal lavage (PPL) would undergo an exploratory laparotomy (EL) with intraoperative cytology, according to Makino’s protocol.^1^ In the following pathway of PPL, we assumed that the laparotomy with a second cytology testing intraoperatively could detect all the OPM that were missed during the PPL.
   2. We assumed that EL can find all the visible peritoneal implants. Considering radiographically occult peritoneal metastases refer to positive cytology and visible peritoneal implants; thus, the false-negative possibility of the strategy of exploratory laparotomy with no cytology testing (ELNC) equals the prevalence of CY1P0 (i.e. positive cytology without visible peritoneal implants).
   3. We assumed that complications did not affect the accuracy of positive peritoneal cytology (PCY) detection.
   4. We assumed non-fatal complications would increase cost but result in no long-term influence on utility.
   5. Fatal complications were assumed not to be observed in the PPL subtree, based on previous reports.1-3
   6. In the decision model, data inputs of transition probabilities were derived from published studies, preferentially meta-analyses or studies with the largest population that similar to our hypothetical population (Table 1). If more than two available estimates were observed, we used pooled estimates derived from meta-analyses using the metamisc package (version 0.2.0) and mada package (version 0.5.9) in R version 3.6.0.
2. Markov models
   1. Model structure and assumption

Each Markov model comprises three health states, i.e., "disease-free survival" (DFS), or "progression-free survival" (PFS), "progressive disease" (PD), and "Death", in which DFS-PD-Death and PFS-PD-Death are used for curative resection Markov model and non-curative palliative resection/ chemotherapy Markov model, respectively. (Figure 1B)

We assumed patients at Disease/Progression-free survival (DFS/PFS) state could stay at DFS/PFS, progress to states of progressive disease (PD) and subsequent death due to the disease of interest, or die from any other causes. Only patients at PD state can die from gastric cancer (GC).

- 1. Calculation of transition probabilities between Markov states

Transition probabilities between these various health states were derived from the Chinese life tables and randomized controlled trials (RCTs) that were suitable to describe the study population in our model.4-8

1) DFS/PFS to Death from life tables

The transition probabilities from DFS/PFS to Death were derived from the age-specific mortality rate due to both all-cause and gastric cancer among the general Chinese population from life tables.4,5 We subtracted age-specific mortality rate due to gastric cancer from age-specific mortality rate due to all causes to calculate the transition rate from DFS/PFS to Death.9 Next, we loaded the age-specific mortality rate into TreeAge software and used function RateToProb to transform the rate into the transition probability from DFS/PFS to Death.

1. Calculation of transition probabilities from clinical trials

In order to calculate the transition probabilities, we selected the corresponding clinical trials that were suitable for the health states and our hypothetical population. In order to choose parameter proportional hazards models to extrapolate and fit Kaplan-Meier curves of the clinical trials, we compared four commonly used parametric survival models to estimate transition probabilities (Table 2).

i. Clinical trials selection

**CLASSIC trial for DFS to PD and** **DFS to DFS**

When calculating the time-dependent transition probabilities from DFS to PD and probabilities from DFS to DFS for curative surgery arm, we used the DFS Kaplan-Meier curves from the CLASSIC trial.7 The CLASSIC trial is a multicentre, randomized phase III trial examing the effect of gastrectomy and adjuvant chemotherapy among the eastern Asian population. The trial is similar to our hypothetical cohort with curative surgery in that the trial and our study both focused on the locally advanced gastric cancer with no evidence of metastatic disease among the eastern Asian population. Besides, our study assumed the age of our hypothetical population to be 56 years old, which is the same as the average age in the CLASSIC trial. Moreover, Disease-free survival was defined as the time from surgery to the date of PD or death in the CLASSIC trial; thus, the DFS Kaplan-Meier curves measured the fraction of the enrolled patients who are free from disease progression or death at a certain time point. Therefore, by making use of DFS Kaplan-Meier curves from the CLASSIC trial and mortality statistics from Chinese life tables, we could subsequently calculate the time-dependent transition probability from DFS to DFS as well as the probability from DFS to PD for curative surgery arm in our model. We also assumed individuals who entered the Markov model of curative surgery begin a six-month course of adjuvant chemotherapy according to the CLASSIC trial.

**REGATTA trial for PFS to PD and PFS to PFS**

To calculate the time-dependent transition probabilities from PFS to PD and PFS to PFS of both non-curative chemotherapy and palliative surgery arm, we used the Kaplan-Meier curves from the REGATTA trial.6 The REGATTA trial, a phase 3 RCT comparing gastrectomy plus chemotherapy with chemotherapy alone for advanced GC patients with a single non-curable factor, is similar to the corresponding scenarios of chemotherapy and palliative surgery in our model. Consequently, we used data from the PFS Kaplan-Meier curves of surgery and chemotherapy arms in the REGATTA trial to calculate the time-dependent transition probabilities of PFS to PD and PFS to PFS in the palliative resection and the chemotherapy Markov models, due to the lack of high-quality evidence on directly comparing palliative gastrectomy plus chemotherapy with chemotherapy alone for PCY metastases.

**COUGAR-02 for PD to Death**

The transition probabilities from PD to Death were derived from clinical trial COUGAR-02.8 COUGAR-02 is a phase 3 randomized controlled trial, focusing on patients with recurrent or metastasis oesophagogastric adenocarcinoma refractory to the first-line chemotherapy. In our model, as DFS patients with curative surgery would receive first-line adjuvant chemotherapy and PFS with the non-curative disease should also undergo chemotherapy of first-line drug, patients with disease progression from both DFS and PFS in our model were refractory to the first-line chemotherapy and therefore were assumed to be similar to the enrolled patients of COUGAR-02.

ii. Selection of parametric distribution

When calculating the time-dependent probability of transition during each Markov model cycle, we first extracted corresponding survival curves from the above clinical trials using Engauge Digitizer version 10.8 software. Next, we compared four commonly used parametric models to fit the selected Kaplan-Meier curves, which included Weibull, exponential, log-logistic, and log-normal. We chose the most reasonable survival distribution function based on clinical rationality, visual fit, and statistical goodness-of-fit using Bayesian information criteria (BIC) and Akaike information criteria (AIC).

We first considered clinical rationality. If the fitted distribution function led to unreasonable survival rates or survival time, the distribution would be excluded. For example, in Figure S1A, the transition probability derived from PFS survival curves P(t) ((P(t)=P_DFStoDFS_+P_DFStoDeath_) in the curative surgery arm between ages 70-74 from the start age of 56 was modelled to be 14.1% and 15.9% according to the log-logistic and log-normal function, respectively. These figures were unreasonably lower than the Chinese life table-derived age-specific probability of dying with a value of 20.4% (approximately equal to P_DFStoDeath_) between ages 70-74 and TreeAge software would produce error messages in this situation.4 Thus, log-logistic and log-normal distributions would not be taken into consideration in this case. Next, the visual inspection of the survival curves was performed where the distributions which were visually far away from the observed Kaplan-Meier curve were excluded. For instance, Figure S1B showed that all curves from four distributions had a similar fit until the 10th month, where about 50% of patients had progressed; however, the exponential model overestimated the disease progression beyond the point. Finally, statistical goodness-of-fit was used to decide the most reasonable survival distribution function according to the minimal value of AIC and BIC among four distributions. The visual fits and statistical fits of CLASSIC, REGATTA, and Cougar-02 K-M curves are presented in Figure S1 and Table S1.

Therefore, the Weibull distribution was selected to fit the transition probability from DFS to PD and PD to Death, while the log-logistic distribution was chosen to calculate transition probability from PFS to PD.

iii. Statistical calculation of transition probability

Statistically, the transition probability P(t) during cycle t (t≥1) is calculated according to the formula P(t) = [S(t-1)-S(t)]/S(t-1), where S(t) is the survivor function of clinical trials.10 According to our model structure (Figure 1B), the following transition probabilities were computed: probabilities from DFS to Death (P_DFStoDeath_), probabilities from DFS to PD (P_DFStoPD_), probabilities of staying at DFS (P_DFStoDFS_), probabilities from PFS to Death (P_PFStoDeath_), probabilities from PFS to PD (P_PFStoPD_), probabilities of staying at PFS (P_PFStoPFS_), probabilities of PD to Death (P_PDtoDeath_), and probabilities of staying at PD (P_PDtoPD_). Based on the definition of DFS and PFS in the CLASSIC and REGATTA trial, the formulae to calculate the transition probability of DFS (or PFS) to DFS (or PFS) and DFS (or PFS) to PD are P_DFStoDFS_ (or P_PFStoPFS_) = 1-P(t), P_DFStoPD_ (or P_PFStoPFS_) = P(t)-P_DFStoDeath_ (or P_DFStoDeath_), in which calculation of P_DFStoDeath_ or P_DFStoDeath_ was explained above. According to the definition of OS in the COUGAR-02 tial, P_PDtoDeath_ is equal to P(t), and the transition probability of PD to PD was calculated by formula P_PDtoPD_=1-P_PDtoDeath_=1-P(t). Based on the above selection, Weibull and log-logistic distribution were chosen to fit Kaplan–Meier curves of the clinical trials in our study. We respectively applied the survival function of Weibull and log-logistic distribution Sw(t) = exp(-λt^γ^) and Sll(t) =1/(1+at^b^) to the calculation of P(t) in different state transition processes. Distribution parameters were first estimated by using a nonlinear least-squares method (nls function from stats package) in R and then loaded into TreeAge software to calculate time-dependent transition probabilities (Table S1).

1. Costs
   1. Annual direct and indirect costs of surgery, chemotherapy, palliative therapy were obtained from a recent national multi-center survey in China (Table1).11 The base-case value of these cost inputs was estimated using the national average values, while the range for these cost inputs was estimated using the values from rural to urban areas.
   2. The costs of adjuvant chemotherapy were calculated based on the regimen, every three weeks for eight cycles, as suggested in an economic evaluation of adjuvant chemotherapy for operable GC in the Chinese population.12,13
   3. The cost of patient surveillance was calculated according to the CSCO national guidelines (Supplemental Table 2).12 All patients were assumed to comply with the guideline recommendations. Patients received follow-up tests every three months in the first two years, followed by once every six months until five years. Each follow-up consists of clinical history query, physical examination, blood chemistry (including CEA and CA19-9) test, Helicobacter pylori (HP) test, performance status assessment, and weight monitoring. Chest, abdominal, and pelvic computed tomography (CT) scans were checked twice-yearly; gastroscopy was performed once a year after resection. The frequency of all follow-up items was reduced to once per year since the fifth year after treatment. The expenditures of each item during follow-up were acquired from the above comprehensive cancer center (Supplemental Table 3).
   4. The costs of surgical complications, including complications related to PPL, laparoscopy, laparotomy, and gastrectomy, were estimated to triple the average surgical costs, according to the study of Luke on the association of hospital costs with complications for gastric adenocarcinoma.14
   5. We did not take the additional costs due to the adverse events related to chemotherapy into account, as it had been included in the chemotherapy cost data based on the previous studies.

1. QALYs
   1. Considering the influence of adjuvant chemotherapy on QALYs, the state after surgery is divided into postoperative adjuvant chemotherapy state and the long-term postoperative state with adjuvant chemotherapy finished. We assumed the utility value of the postoperative adjuvant chemotherapy state was 0.68 (within six months after surgery), and the value of long-term postoperative state with the accomplishment of adjuvant chemotherapy was 0.81 (more than six months), according to a Chinese health economic study involving adjuvant chemotherapy.15
   2. QALYs for recurrent or progressive GC with palliative chemotherapy were 0.40, derived from a cross-sectional survey on utility weights for GC-related health states in a Korean general population which is similar to the Chinese.16
   3. QALYs were assigned as 0.66 for metastasis GC with chemotherapy, and 0.54 for palliative surgery plus chemotherapy, both of which came from Li’s cost-effectiveness study among GC patients.17
2. Sensitivity Analyses
   1. When conducting probabilistic sensitivity analyses, we estimated the parameters of the specific distributions with means and confidence intervals (or standard deviation) using TreeAge Pro 2019 (TreeAge Software Inc., Williamstown, MA, USA) and Parameter Solver Version 3.0.0 (Department of Biostatistics of MD Anderson Cancer Center, Houston, TX, USA).
   2. If ranges or standard deviation were not available from published literature, the range for model inputs were assumed to vary within a range of 50-150% base value in the sensitivity analysis.

Figure S1


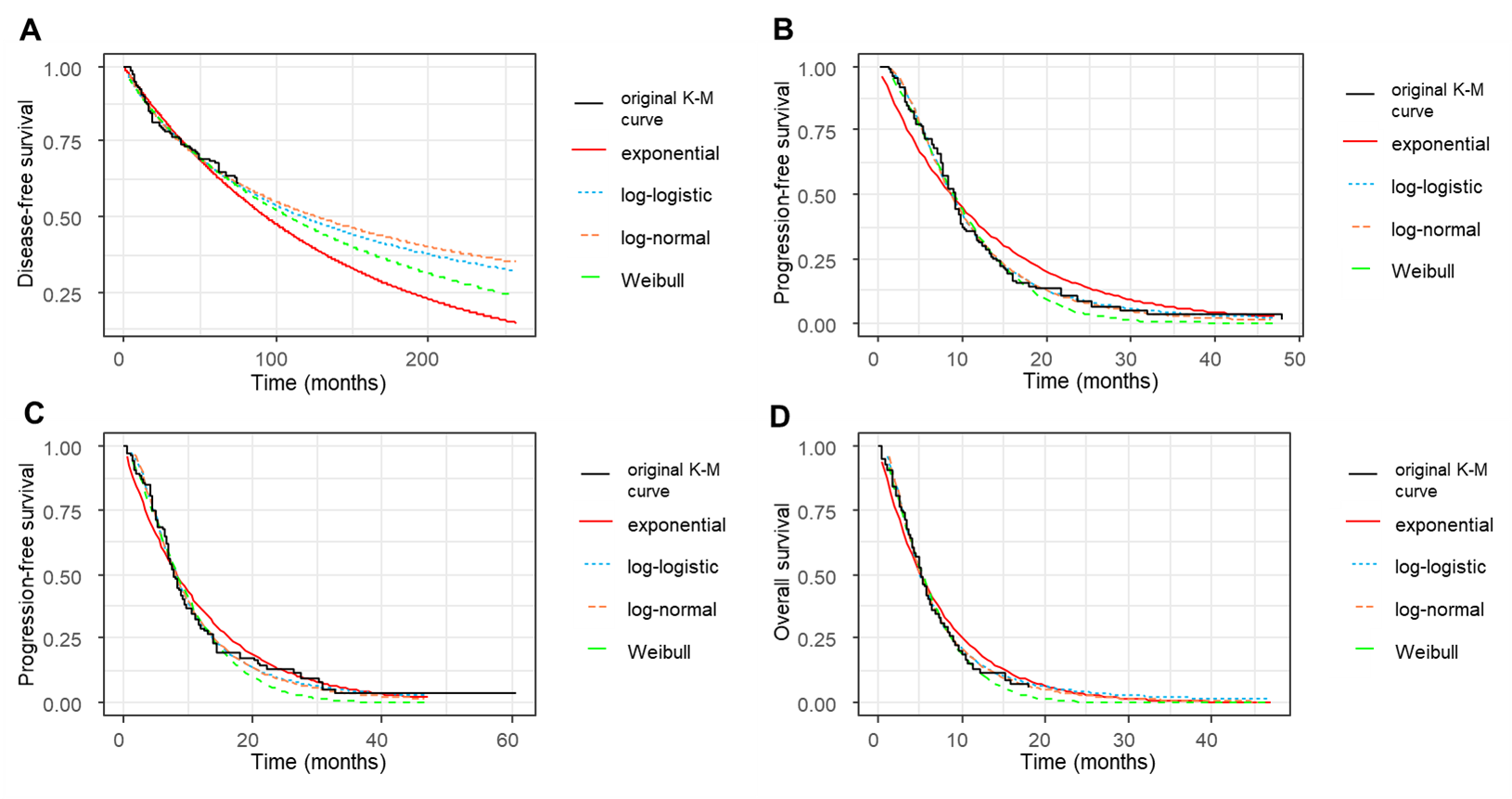


Figure S1. Fitting and extrapolation of Kaplan-Meier curve of randomized controlled trials. **A** for DFS curve of surgery with adjuvant chemotherapy arm in CLASSIC trial; **B** for PFS curve of chemotherapy arm of REGATTA trial; **C** for PFS non-curative palliative surgery arm of REGATTA trial; **D** for OS of docetaxel arm of COUGAR-02 study. Abbreviations: DFS, disease-free survival OS, overall survival; PFS, progression-free survival.

Tables

Table S1 Summary of distribution for fitting and extrapolation of clinical trials.

| Distribution† | Parameter value (SD) | AIC | BIC |
| --- | --- | --- | --- |
| DFS of surgery plus adjuvant chemotherapy arm of CLASSIC trial | | | |
| Weibull | λ=0.0132 (0.0010),  γ=0.8454 (0.0192) | -542.44 | -534.40 |
| Exponential | λ=0.0074 (0.0001) | -493.14 | -487.77 |
| Log-logistic | a=0.0105 (0.0001)  b=0.9549 (0.0197) | -564.86 | -556.81 |
| Log-normal | μ=4.8468 (0.0233)  δ=1.8403 (0.0307) | -604.54 | -596.49 |
| PFS of chemotherapy arm of REGATTA trial | | | |
| Weibull | λ=0.0218 (0.0012),  γ=1.4907 (0.0234) | -613.09 | -604.10 |
| Exponential | λ=0.0800 (0.0001) | -327.65 | -321.65 |
| Log-logistic | a=0.0069 (0.0005)  b=2.3024 (0.0297) | -667.54 | -658.55 |
| Log-normal | μ=2.1619 (0.0057)  δ=0.7301 (0.0098) | -638.29 | -629.29 |
| PFS of palliative surgery arm of REGATTA trial | | | |
| Weibull | λ=0.0366 (0.0023),  γ=1.3827 (0.0234) | -544.86 | -535.85 |
| Exponential | λ=0.0835 (0.0014) | -405.42 | -399.42 |
| Log-logistic | a=0.0142 (0.0008)  b=2.0360 (0.0271) | -677.06 | -668.05 |
| Log-normal | μ=2.0912 (0.0063)  δ=0.8228 (0.0121) | -632.42 | -623.41 |
| OS of docetaxel arm of COUGAR-02 trial | | | |
| Weibull | λ=0.0654 (0.0017),  γ=1.3663 (0.0128) | -748.15 | -739.16 |
| Exponential | λ=0.0835 (0.0014) | -449.20 | -443.21 |
| Log-logistic | a=0.0142 (0.0008)  b=2.0360 (0.0271) | -719.49 | -710.50 |
| Log-normal | μ=2.0912 (0.0063)  δ=0.8228 (0.0121) | -683.68 | -674.69 |

Abbreviations: DFS, disease-free survival; PFS, progression-free survival; PD, progressive disease; OS, overall survival.

† The survival function of Weibull, exponential, log-logistic, and log-normal distribution respectively is: 𝑆w(𝑡) = exp(-λt^γ^), 𝑆e(𝑡) = exp(-λ^t^), 𝑆ll(𝑡) =1/(1+at^b^), and 𝑆ln(𝑡) = 1−𝜑[(ln𝑡 −𝜇)/𝜎](where 𝜑 is a cumulative distribution function from normal distribution.)

Table S2 Follow‑up visits for advanced gastric cancer patients after radical resection according to the Chinese Society of Clinical Oncology (CSCO) guideline.

a. Contents and frequency of Follow‑up visits

| Contents | Frequency |
| --- | --- |
| Clinical history, physical examination, blood chemistry (including CEA and CA19‑9), HP detection, performance status monitoring, weight monitoring, chest, abdominal, and pelvic CT every six months (especially for those with abnormal CEA levels) | Once every three months in the first two years, followed by once every six months until five years |
| Chest, abdominal, and enhanced pelvic CTs; Gastroscopy (recommended once a year) | Once every year for more than five years after treatment |

b. Item and corresponding costs of Follow‑up visits

| Item | Cost, US$ |
| --- | --- |
| Blood chemistry (including CEA and CA19‑9)  CEA | 20  6 |
| CA19-9 | 14 |
| Outpatient follow-up (containing a collection of clinical history, physical examination, and performance status and weight monitoring) | 12 |
| Helicobacter pylori (HP) | 37 |
| CT scans | 180 |
| Chest CT | 20 |
| Abdominal CT | 40 |
| Pelvic CT | 20 |
| Contrast enhancement agent | 32 |
| CT films | 44 |
| Others (such as injection fees, nursing, etc.) | 24 |
| Gastroscopy | 278 |
| Endoscopy preparation (including drug, laboratory test, nursing, etc.) | 136 |
| Endoscopy (including endoscopy, biopsy and pathology) | 142 |

Abbreviations: CT computed tomography.

Table S3 Parameter Thresholds at Which PPL is cost-effective

| Parameter^†^ | Base case | Range | Threshold |
| --- | --- | --- | --- |
| Perioperative mortality of EL | 1.5% | 0-3% | 1.06% |
| prevalence of OPM | 23.4% | 8.5-59.6% | 31.03% |
| Sensitivity of LPL | 84.6% | 74.7-91.8% | 73.29% |
| Utility of postgastrectomy | 0.81 | 0.648-0.972 | 0.51 |
| Cost of LPL | 2213 | 1107.5-3319.5 | 4343 |
| Cost of chemotherapy | 5256 | 3169-9728 | 22953 |
| Perioperative mortality of surgery | 2.7% | 0.3-7.5% | 18.05% |
| Sensitivity of PPL | 77.0% | 75.0-83.3% | Never |
| Cost of PPL | 246 | 123-369 | Never |

Abbreviations: EL, exploratory laparotomy; LPL, laparoscopic peritoneal lavage; OPM, occult peritoneal metastases; PPL, percutaneous peritoneal lavage.

† The select parameters are stacked in a decreasing order of effect each input has on the outcome according to the order of Tornado diagrams.

References:

1 Tomoki M, Yoshiyuki F, Shuji T, et al. The utility of pre-operative peritoneal lavage examination in serosa-invading gastric cancer patients. *Surgery*. 2010; **148**(1): 96-102

2 Kimberly KN, Roxanne RR, Kimberly TJ, et al. Experience with over 2500 diagnostic peritoneal lavages. *Injury*. 2000; **31**(7): 479-482

3 Pak LM, Coit DG, Eaton AA, et al. Percutaneous Peritoneal Lavage for the Rapid Staging of Gastric and Pancreatic Cancer. *Ann Surg Oncol*. 2017; **24**(5): 1174-1179

4 WHO Life tables for China. Available at: http://apps.who.int/gho/data/?theme=main&vid=60340. Accessed 30th June 2019. In

5 Lei Y, Rongshou Z, Ning W, et al. Incidence and mortality of stomach cancer in China, 2014. *Chin J Cancer Res*. 2018; **30**(3): 291-298

6 Fujitani K, Yang HK, Mizusawa J, et al. Gastrectomy plus chemotherapy versus chemotherapy alone for advanced gastric cancer with a single non-curable factor (REGATTA): a phase 3, randomised controlled trial. *Lancet Oncol*. 2016; **17**(3): 309-318

7 Sung HN, Sook RP, Han-Kwang Y, et al. Adjuvant capecitabine plus oxaliplatin for gastric cancer after D2 gastrectomy (CLASSIC): 5-year follow-up of an open-label, randomised phase 3 trial. *Lancet Oncol*. 2014; **15**(12): 1389-1396

8 Ford HER, Andrea M, Bridgewater JA, et al. Docetaxel versus active symptom control for refractory oesophagogastric adenocarcinoma (COUGAR-02): an open-label, phase 3 randomised controlled trial. *Lancet Oncol*. 2014; **15**(1): 78-86

9 Briggs A, Sculpher M. An Introduction to Markov Modelling for Economic Evaluation. *Pharmacoeconomics*. 1998; **13**(4): 397-409

10 Briggs A, Sculpher M, Claxton K. Decision Modelling for Health Economic Evaluation.1st ed. Northants, UK: Oxford University Press; 2006

11 Zhixun Y, Hongmei Z, Ruyi X, et al. Annual cost of illness of stomach and esophageal cancer patients in urban and rural areas in China: A multi-center study. *Chin J Cancer Res*. 2018; **30**(4): 439-448

12 Wang FH, Shen L, Li J, et al. The Chinese Society of Clinical Oncology (CSCO): clinical guidelines for the diagnosis and treatment of gastric cancer. *Cancer Commun (Lond)*. 2019; **39**(1): 10

13 He J, Wen F, Yin X, et al. Cost analysis of S1 and XELOX as adjuvant therapy for gastric cancer. *Anticancer Drugs*. 2013; **24**(7): 754-758

14 Luke VS, Renee LG, Geoffrey CS, et al. Association of Hospital Costs With Complications Following Total Gastrectomy for Gastric Adenocarcinoma. *Jama Surg*. 2017; **152**(10): 953-958

15 Chongqing T, Liubao P, Xiaohui Z, et al. Economic evaluation of first-line adjuvant chemotherapies for resectable gastric cancer patients in China. *Plos One*. 2013; **8**(12):

16 Lee HJ, Ock M, Kim KP, Jo MW. Estimation of population-based utility weights for gastric cancer-related health states. *Patient Preference & Adherence*. 2018; **12**(909-918

17 Li. K, Cannon. JGD, Jiang. SY, et al. - Diagnostic staging laparoscopy in gastric cancer treatment: A cost-effectiveness analysis. *- J Surg Oncol*. 2018; **- 117**(- 6): 1288-1296
